# Exploring Hypertrophic Cardiomyopathy Biomarkers through Integrated Bioinformatics Analysis: Uncovering Novel Diagnostic Candidates

**DOI:** 10.1155/2024/4639334

**Published:** 2024-07-04

**Authors:** Guanmou Li, Dongqun Lin, Xiaoping Fan, Bo Peng

**Affiliations:** ^1^ Zhujiang Hospital of Southern Medical University, Guangzhou 510120, Guangdong, China; ^2^ Department of Cardiovascular Surgery Guangdong Provincial Hospital of Chinese Medicine The Second Affiliated Hospital of Guangzhou University of Chinese Medicine The Second Clinical College of Guangzhou University of Chinese Medicine, Guangzhou 510120, Guangdong, China; ^3^ State Key Laboratory of Dampness Syndrome of Chinese Medicine, Guangzhou 510120, Guangdong, China; ^4^ Guangdong Provincial Key Laboratory of TCM Emergency Research, Guangzhou 510120, Guangdong, China

## Abstract

HCM is a heterogeneous monogenic cardiac disease that can lead to arrhythmia, heart failure, and atrial fibrillation. This study aims to identify biomarkers that have a positive impact on the treatment, diagnosis, and prediction of HCM through bioinformatics analysis. We selected the GSE36961 and GSE180313 datasets from the Gene Expression Omnibus (GEO) database for differential analysis. GSE36961 generated 6 modules through weighted gene co-expression network analysis (WGCNA), with the green and grey modules showing the highest positive correlation with HCM (green module: cor = 0.88, *p* = 2*e* − 48; grey module: cor = 0.78, *p* = 4*e* − 31). GSE180313 generated 17 modules through WGCNA, with the turquoise module exhibiting the highest positive correlation with HCM (turquoise module: cor = 0.92, *p* = 6*e* − 09). We conducted GO and KEGG pathway analysis on the intersection genes of the selected modules from GSE36961 and GSE180313 and intersected their GO enriched pathways with the GO enriched pathways of endothelial cell subtypes calculated after clustering single-cell data GSE181764, resulting in 383 genes on the enriched pathways. Subsequently, we used LASSO prediction on these 383 genes and identified RTN4, COL4A1, and IER3 as key genes involved in the occurrence and development of HCM. The expression levels of these genes were validated in the GSE68316 and GSE32453 datasets. In conclusion, RTN4, COL4A1, and IER3 are potential biomarkers of HCM, and protein degradation, mechanical stress, and hypoxia may be associated with the occurrence and development of HCM.

## 1. Background

Hypertrophic cardiomyopathy (HCM) is a heterogeneous monogenic cardiac disease [1] that is a significant cause of arrhythmic sudden death, heart failure, and atrial fibrillation (with thromboembolic stroke) [2–4]. The estimated prevalence of HCM in the general adult population is approximately 0.2% (1 : 500) [5]. HCM is an autosomal dominant genetic disease, and it has been confirmed that 11 genes play crucial roles in HCM; any mutation in these genes can lead to the occurrence of HCM. Among these 11 key genes, three genes dominate mutations. In HCM, 70% of gene mutations are related to *β*-myosin heavy chain (MYH7) and myosin-binding protein C (MYBPC3) [6]. Troponin T (TNNT2) and several other genes each contribute to 5% or fewer cases. Other genes are also expressed in HCM, such as cardiac *α*-myosin heavy chain (myosin 6), titin (TTN), muscle LIM protein (CSRP3), telethonin (TCAP), and junctophilin 2 (JPH2) [7].

However, the current findings of gene mutations are not sufficient to explain certain morphological changes that occur in HCM, such as mitral valve enlargement [8], microvascular anomalies [9], and segmental left ventricular hypertrophy [10]. Although we understand the crucial role of genes in HCM, there are currently no gene therapies available for clinical use. Even though a team has demonstrated the successful use of AAV9-mediated functional MYBPC3 cDNA gene replacement in mouse and human pluripotent stem cell-derived cardiac myocytes to correct myofiber mutations, there is still a distance to go before clinical application [11]. We need to explore new genes that affect the occurrence and development of HCM and further elucidate the pathological processes involved in triggering and exacerbating HCM.

**Figure 1:**
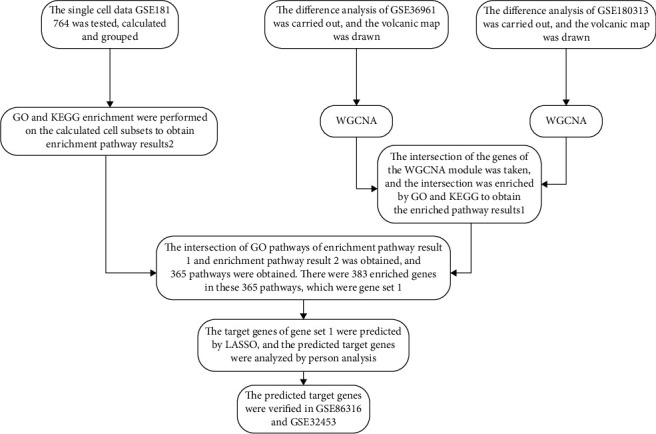
Workflow for identifying biomarkers in HCM.

Our research data come from the Gene Expression Omnibus (GEO) database. We used weighted gene co-expression network analysis (WGCNA) to select module genes and machine learning (LASSO) to predict potential biomarkers affecting HCM (Figure 1). Our study aims to promote a biological perspective and distinguish potential biomarkers for the diagnosis and treatment of HCM.

## 2. Materials and Methods

### 2.1. Materials

The materials and methods dataset for GSE36961 provided by Hebl et al., GSE89714 provided by Li et al., GSE180313 provided by Ranjbarvaziri et al., GSE68316 provided by Wu et al., and GSE32453 provided by Gennebäck et al. were obtained from the GEO database (https://www.ncbi.nlm.nih.gov/geo/). Gene expression profiles were generated using GPL15389 (Illumina HumanHT-12 V3.0 expression beadchip), GPL11154 (Illumina HiSeq 2000), GPL24676 (Illumina NovaSeq 6000), GPL20113 (CapitalBio Human LncRNA Microarray v2.0), and GPL6104 (Illumina humanRef-8 v2.0 expression beadchip). The GSE36961 dataset includes 106 disease samples and 38 normal samples, GSE89714 includes 5 disease samples and 4 healthy samples, GSE180313 includes 13 disease samples and 7 healthy samples, GSE68316 includes 7 disease samples and 5 healthy samples, and GSE32453 includes 8 disease samples and 5 healthy samples. The single-cell data of HCM samples in the GSE181764 dataset were provided by Codden et al. (GSM5511031 2890). Statistical analysis was performed using R software (version 4.2.0).

### 2.2. Data Preprocessing and Differential Analysis

In the GSE36961, GSE180313, GSE32453, and GSE68316 datasets, after removing invalid probes and missing values using the “stringr” algorithm and selecting the median as the gene expression level, genes with multiple probes for the same gene were normalized. The “limma” algorithm was then used to identify differentially expressed genes (DEGs) in the GSE36961 and GSE180313 datasets. |log2FC| (fold change) greater than 0.37851 (1.3-fold difference) and adjusted *P* value (adj. *P*. Val.) < 0.05 were considered statistically significant. Finally, differential analysis results were used to generate volcano plots using R and the Sangerbox website (http://sangerbox.com/home.html).

### 2.3. WGCNA

The “WGCNA” package in R software was used to construct gene co-expression networks to explore gene expression and interactions in HCM samples. Initially, to establish a scale-free network in the GSE36961 and GSE180313 datasets, different soft-thresholding powers (*R*2 = 0.9 and soft-thresholding power beta = 13, *R*2 = 0.8 and soft-thresholding power beta = 5) were chosen based on the data distribution characteristics of different datasets. Subsequently, a topological overlap matrix (TOM) was used to establish network interconnectedness, and gene modules were identified based on hierarchical clustering. The first principal component of each gene module was determined as the module eigengene (ME). The correlation between clinical characteristics and MEs was examined, and the module most highly correlated with HCM was identified as the key module. Gene significance (GS) was used to identify gene-trait relationships, and module membership (MM) represented the correlation between MEs and gene expression profiles. Genes with high GS and MM in the key module were highly associated with clinical features. A threshold of MM > 0.6 and GS > 0.6 was used for gene selection, and the intersection of genes in the modules most highly correlated with HCM in GSE36961 and GSE180313 was determined using the jvenn website (https://jvenn.toulouse.inrae.fr/app/index.html) and then subjected to GO and KEGG analysis in the Sangerbox website, producing enrichment result 1.

### 2.4. Clustering of Single-Cell Data

The Seurat package in R was used for data normalization and integration to reduce batch effects. Quality control, visualization, normalization of highly variable genes, and PCA calculation, clustering, and dimension reduction using UMAP and t-SNE methods were performed. The expression of the top 60 marker genes in each cluster was compared on the Cell Marker website (http://bio-bigdata.hrbmu.edu.cn/CellMarker/) to determine the cell types of each cluster and complete cell type annotations. GO and KEGG analysis was then conducted on the four obtained clusters, producing enrichment result 2.

### 2.5. LASSO Prediction of Biomarkers and Key Gene Validation

The intersection of pathways in enrichment results 1 and 2 was identified, and the genes on these intersecting pathways were extracted and their expression profiles were obtained from the GSE89714 dataset. The “glmnet” package in R software was used to build a LASSO model based on the gene expression profiles of hub genes. The smallest error (lambda.min) and one standard error of the smallest error (lambda.1se) were used as references to identify variables in the model, and two appropriate lambda values were selected. Subsequently, model fitting and cross-validation were performed to obtain regression coefficients under the optimal lambda in feature selection. Genes corresponding to nonzero coefficients were extracted from the regression coefficients. The expression profiles of the predicted genes by LASSO in the GSE89714 dataset were extracted, and Pearson analysis was performed using the Sangerbox website (http://sangerbox.com/home.html). Additionally, GSE68316, containing 7 HCM patients and 5 human myocardial tissue samples on the GPL20113 platform, and GSE32453, containing 8 HCM patients and 5 human myocardial tissue samples on the GPL6104 platform, were retrieved from the GEO database and used as validation datasets. Student's *t*-test was used to compare the expression variance of key genes between the HCM group and the control group, and expression graphs for the key genes were plotted using the Sangerbox website (http://sangerbox.com/home.html). Furthermore, the Gaussian Naive Bayes algorithm and support vector machine (SVM) algorithm in R software were used to predict whether the key genes played a crucial role in HCM, and demographic information (age and gender) of the key genes was plotted to form a baseline table in the GSE36961 dataset.

## 3. Results

### 3.1. Identification between HCM and Control Groups

**Figure 2:**
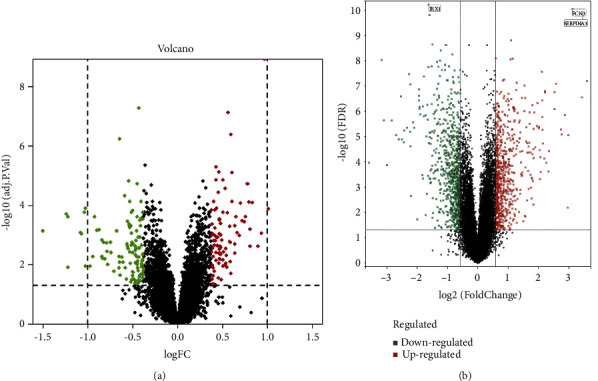
Differential analysis results. (a) Volcano plot of differential genes in GSE36961. (b) Volcano plot of differential genes in GSE180313.

In GSE36961, a total of 206 DEGs, including 113 upregulated genes and 93 downregulated genes, were identified between HCM patients and the control group. In GSE180313, a total of 2919 DEGs, including 1505 upregulated genes and 1414 downregulated genes, were identified between HCM patients and the control group. The results were visualized using volcano plots, with each point corresponding to a gene (Figures 2(a) and 2(b)).

**Figure 3:**
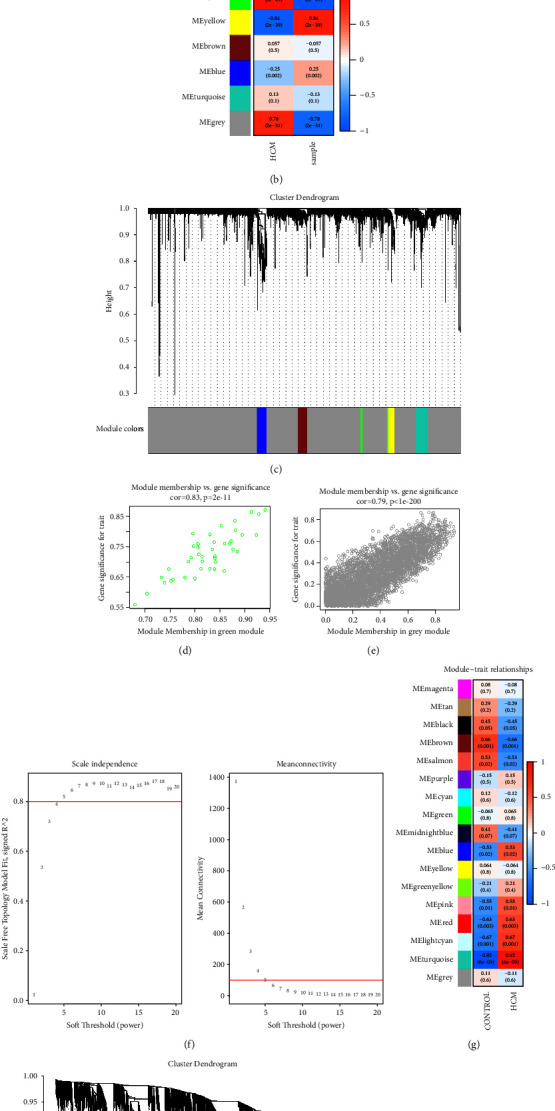
WGCNA analysis result. (a) Soft threshold analysis of GSE36961. Each power corresponds to scale independence and average connectivity. We selected an R cutoff of 0.89 and a soft threshold of 13 (red horizontal line) to construct a scale-free network topology. (b) Module-trait relationships in GSE36961. The numbers in the colored grids represent the correlation and their *p* values. Red indicates positive correlation, and blue indicates negative correlation. (c) Cluster dendrogram of GSE36961. Each branch represents one gene, and each color at the bottom represents a co-expression module. (d) Scatter plot of module membership and gene significance in the green module of GSE36961. (e) Scatter plot of module membership and gene significance in the grey module of GSE36961. (f) Soft threshold analysis of GSE180313. Each power corresponds to scale independence and average connectivity. We selected an R cutoff of 0.8 and a soft threshold of 5 (red horizontal line) to construct a scale-free network topology. (g) Module-trait relationships in GSE180313. The numbers in the colored grids represent the correlation and their *p* values. Red indicates positive correlation, and blue indicates negative correlation. (h) Cluster dendrogram of GSE180313. Each branch represents one gene, and each color at the bottom represents a co-expression module. (i) Scatter plot of module membership and gene significance in the turquoise module of GSE180313.

*Construction of WGCNA and Identification of Hub Gene*. We performed WGCNA to detect the coexpression networks of all genes in GSE36961 and GSE180313 to analyze the coverage. In GSE36961, we chose 13 as the soft threshold to construct a scale-free network topology (Figure 3(a)). Subsequently, a gene co-expression network was constructed based on hierarchical clustering, generating 6 modules (Figure 3(c)). We also conducted correlation analysis of the modules with clinical traits and phenotypes. The green and grey modules showed the highest positive correlation with HCM (green module: cor = 0.88, *p* = 2*e* − 48; grey module: cor = 0.78, *p* = 4*e* − 31) (Figure 3(b)). Subsequently, we focused on further investigating the green and grey modules, which exhibited the maximum absolute correlation across all modules.  Figure 3(d) shows the correlation analysis between MM and GS in the green module, with a correlation value of 0.83 (*p* value = 2*e* − 11).  Figure 3(e) shows the correlation analysis between MM and GS in the grey module, with a correlation value of 0.79 (*p* value <1*e* − 200). The green and grey modules together comprised 3261 genes. In GSE180313, we chose 5 as the soft threshold for constructing a scale-free network topology (Figure 3(f)). Subsequently, a gene co-expression network was constructed based on hierarchical clustering, generating 17 modules (Figure 3(h)). We also conducted correlation analysis of the modules with clinical traits and phenotypes. The turquoise module showed the highest positive correlation with HCM (turquoise module: cor = 0.92, *p* = 6*e* − 09) (Figures 3(d), 3(e) and 3(g)). Subsequently, we further studied the turquoise module, which exhibited the maximum absolute correlation across all modules.  Figure 3(i) shows the correlation analysis between MM and GS in the turquoise module, with a correlation value of 0.91 (*p* value < 1*e* − 200). The turquoise module comprised 1539 genes.

**Figure 4:**
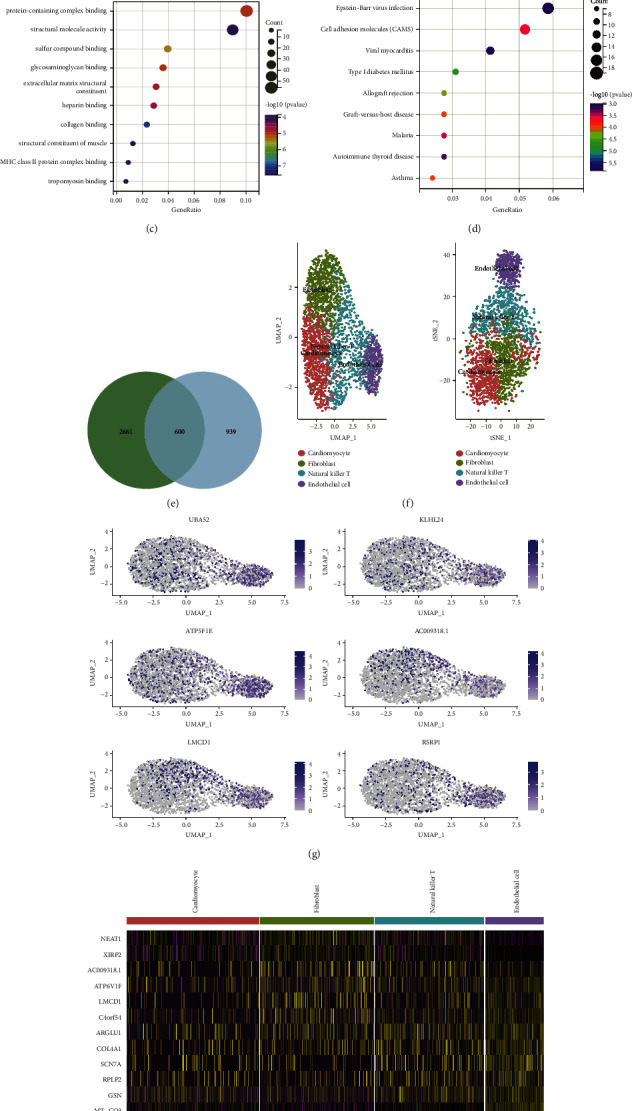
(a) GO BP enrichment diagram of the module intersection genes in WGCNA. (b) GO CC enrichment diagram of the module intersection genes in WGCNA. (c) GO MF enrichment diagram of the module intersection genes in WGCNA. (d) KEGG enrichment diagram of the module intersection genes in WGCNA. (e) Intersection of genes from the green and grey modules in GSE36961 and genes from the turquoise module in GSE180313. (f) UMAP and TSNE diagram of GSE181764. (g) UMAP diagram of the top 6 highly variable genes in GSE181764. (h) Heatmap of the top 10 highly variable genes in 4 cell subtypes of GSE181764 distribution.

Through WGCNA, the intersection of 3261 genes from the green and grey modules selected in GSE36961 and 1539 genes selected in GSE180313 resulted in 600 shared genes (Figure 4(e)). GO and KEGG analysis of these 600 genes showed their involvement in the process of system development (Figure 4(a)) and extracellular region (Figure 4(b)) in GO cellular component analysis, participation in protein-containing complex binding (Figure 4(c)) in GO molecular function analysis, and involvement in the process of phagosome (Figure 4(d)) in GO biological process analysis. The pathways analyzed and the genes enriched in those pathways were summarized as enrichment result 1.

**Figure 5:**
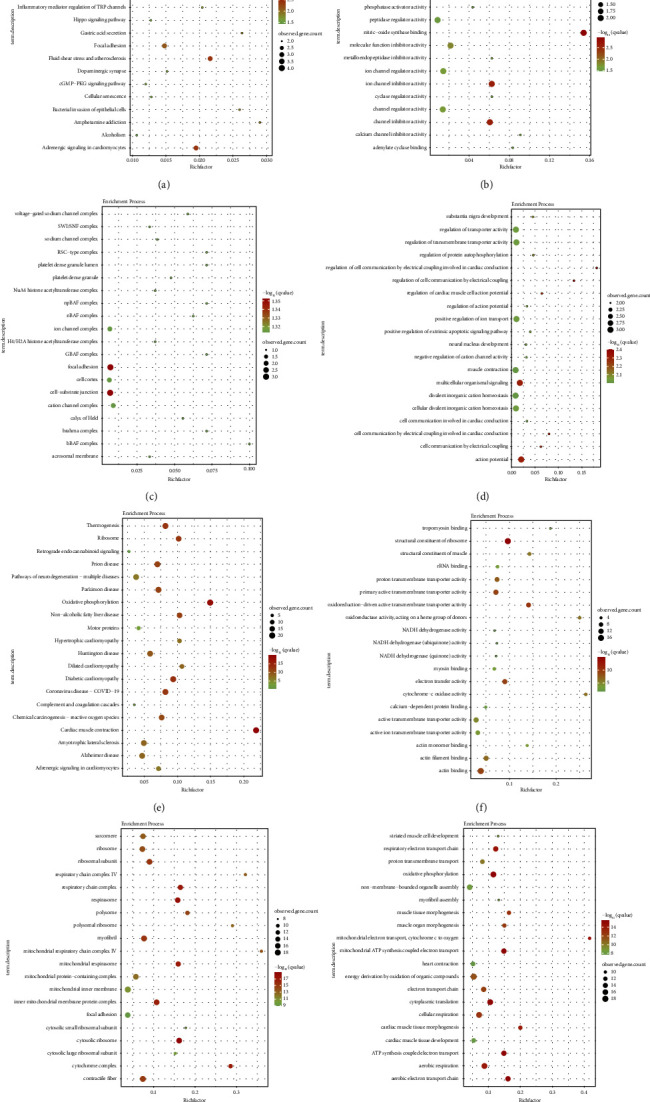
GO and KEGG enrichment diagrams of cardiomyocytes and fibroblasts in GSE181764. (a) KEGG enrichment diagram of cardiomyocytes. (b) GO MF enrichment diagram of cardiomyocytes. (c) GO CC enrichment diagram of cardiomyocytes. (d) GO BP enrichment diagram of cardiomyocytes. (e) KEGG enrichment diagram of fibroblasts. (f) GO MF enrichment diagram of fibroblasts. (g) GO CC enrichment diagram of fibroblasts. (h) GO BP enrichment diagram of fibroblasts.

*Calculation and clustering of single cells*. Quality inspection, PCA dimension reduction, UMAP and t-SNE calculation, and clustering were performed for the HCM sample GSM5511031 2890 in GSE181764 (Figure 4(f)), resulting in the identification of 4 cell subtypes: cardiomyocytes, fibroblasts, natural killer T cells, and endothelial cells. Subsequently, the differential expression of the top 6 highly variable genes in UMAP space for these 4 cell subtypes was analyzed (Figure 4(e)). Additionally, the expression analysis of the top 10 highly variable genes in these 4 cell subtypes was conducted (Figure 4(h)). Marker genes were then separately identified for the 4 cell subtypes, and GO and KEGG analysis was performed. For cardiomyocytes, KEGG analysis showed the involvement of marker genes in the process of viral myocarditis (Figure 5(a)), GO MF analysis showed their involvement in voltage-gated sodium channel activity (Figure 5(b)), GO CC analysis showed their involvement in the voltage-gated sodium channel complex (Figure 5(c)), and GO BP analysis showed their involvement in substantia nigra development (Figure 5(d)). For fibroblasts, KEGG analysis showed the involvement of marker genes in thermogenesis (Figure 5(e)), GO MF analysis showed their involvement in tropomyosin binding (Figure 5(f)), GO CC analysis showed their involvement in the sarcomere (Figure 5(g)), and GO BP analysis showed their involvement in striated muscle cell development (Figure 5(h)).

**Figure 6:**
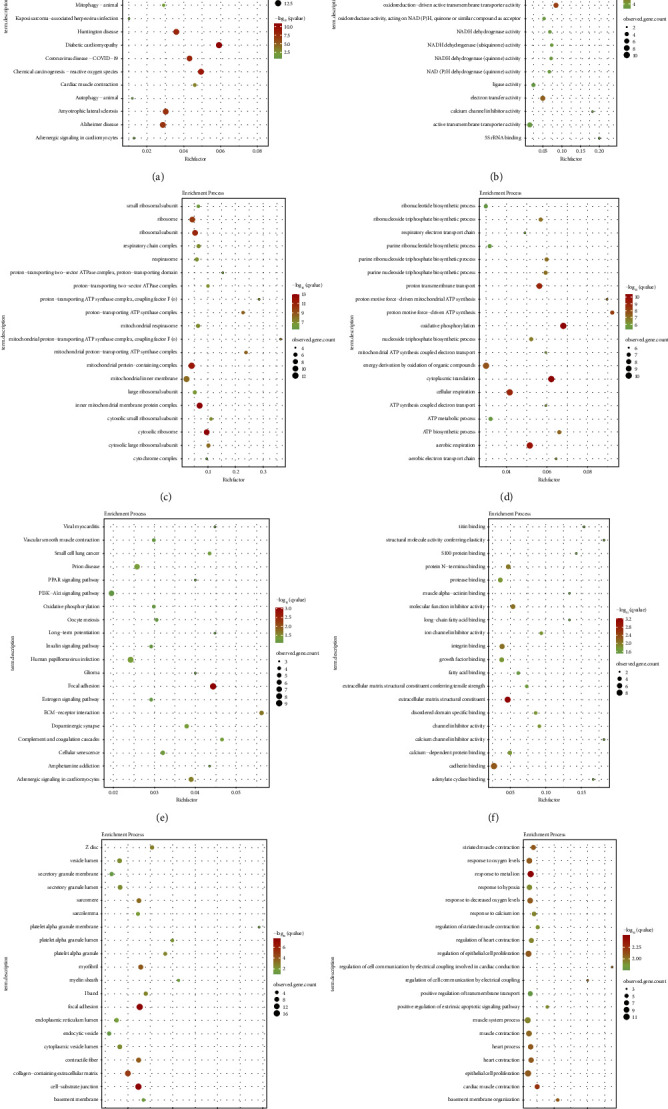
Enrichment maps of natural killer T cells and endothelial cells in GSE181764. (a) KEGG enrichment map of natural killer T cells. (b) GO MF enrichment map of natural killer T cells. (c) GO CC enrichment map of natural killer T cells. (d) GO BP enrichment map of natural killer T cells. (e) KEGG enrichment map of endothelial cells. (f) GO MF enrichment map of endothelial cells. (g) GO CC enrichment map of endothelial cells. (h) GO BP enrichment map of endothelial cells.

In natural killer T cells, KEGG analysis showed the involvement of marker genes in the ubiquitin-mediated proteolysis process (Figure 6(a)), GO MF analysis showed their involvement in ubiquitin protein ligase binding (Figure 6(b)), GO CC analysis showed their involvement in the small ribosomal subunit (Figure 6(c)), and GO BP analysis showed their involvement in the ribonucleotide biosynthetic process (Figure 6(d)). In endothelial cells, KEGG analysis showed the involvement of marker genes in the viral myocarditis process (Figure 6(e)), GO MF analysis showed their involvement in titin binding (Figure 6(f)), GO CC analysis showed their involvement in the Z disc (Figure 6(g)), and GO BP analysis showed their involvement in the striated muscle contraction process (Figure 6(h)). The pathways analyzed and the genes enriched in those pathways for the endothelial cell subtype were summarized as enrichment result 2.

**Figure 7:**
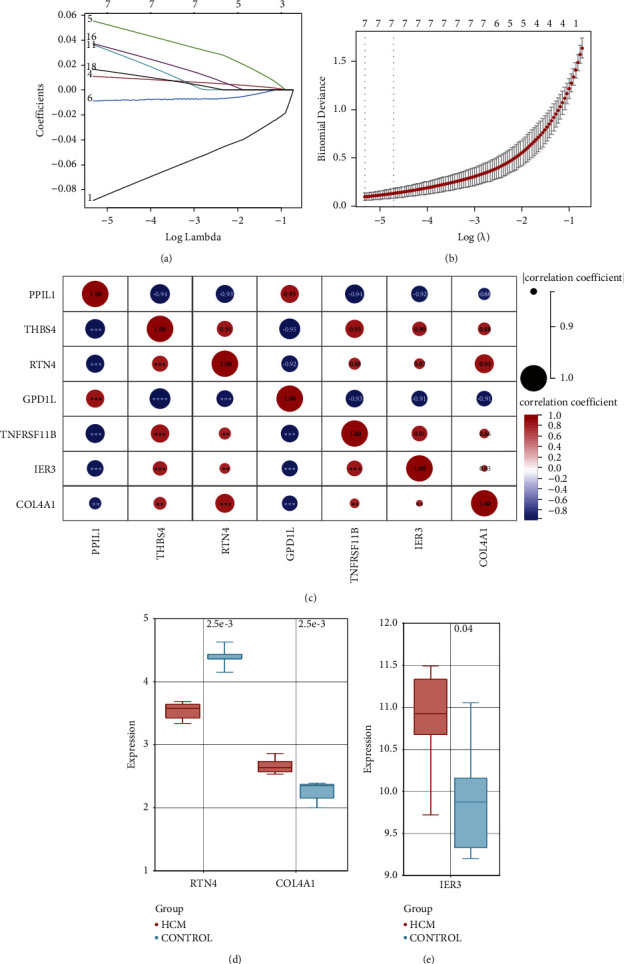
(a) Cross-validation of the 7 predicted genes in the fitted model. (b) Optimal selection in the LASSO model. The dashed line on the left represents lambda.min, and the dashed line on the right represents lambda.1se. (c) Pearson analysis of the 7 predicted genes. (d) Differential expression of RTN4 and COL4A1 in the validation dataset GSE68316. (e) Differential expression of IER3 in the validation dataset GSE32453.

**Figure 8:**
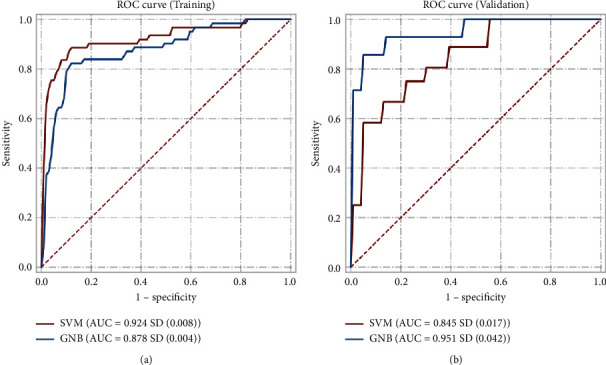
Highly effective translator (GNB) and support vector machine (SVM) algorithms predict whether RTN4, IER3, and COL4A1 play a key role in HCM. (a) AUC curve for the training set. (b) AUC curve for the test set.

*LASSO prediction of biomarkers and validation of key genes*. The intersection of enriched pathways from Result 1 and Result 2 was obtained, and the genes on the intersected pathways were extracted. After removing duplicate values, a total of 365 intersected pathways and 383 genes were obtained. The expression profiles of these 383 genes were extracted from GSE89714, and a LASSO model was then established for model fitting and cross-validation to predict biomarkers. Finally, 7 was selected as the best regression coefficient (Figure 7(a)), and the cross-validation plot of these 7 genes (PPIL1, THBS4, RTN4, GPD1L, TNFRSF11B, IER3, and COL4A1) was constructed (Figure 7(b)). Subsequently, Pearson's analysis was performed on these 7 predicted genes. The correlation coefficient between RTN4 and COL4A1 was 0.94, the correlation coefficient between RTN4 and IER3 was 0.87, and the correlation coefficient between IER3 and COL4A1 was 0.83, indicating a strong correlation (Figure 7(c)). RTN4 and COL4A1 were validated in GSE68316, and the *p* values were found to be less than 0.05, showing significant differences between the HCM group and the normal group (Figure 7(d)). IER3 was validated in GSE32453, and the *p* value was found to be less than 0.05, also showing a significant difference between the HCM group and the normal group (Figure 7(e)). Finally, Gaussian Naive Bayes and support vector machine (SVM) algorithms were used to predict whether RTN4, IER3, and COL4A1 play key roles in HCM. The results show that the AUC values of the training sets of RTN4, IER3, and COL4A1 in the Gaussian Naive Bayes algorithm (GNB) and the support vector machine (SVM) algorithm are 0.878 and 0.924, respectively. The AUC values of the test sets of RTN4, IER3, and COL4A1 in the Gaussian Naive Bayes algorithm (GNB) and the support vector machine (SVM) algorithm are 0.951 and 0.845, respectively. It can be seen that the model prediction effect of RTN4, IER3, and COL4A1 is good (Figure 8). Furthermore, demographic information (age and gender) baseline analysis of RTN4, IER3, and COL4A1 showed that age and gender were not related to the expression of these three genes (*p* > 0.05), indicating the robustness of these biomarkers in predicting HCM (see Supplementary Table 1).

## 4. Discussion

In this study, the gene profiles of HCM patients and a healthy control group were comprehensively analyzed for potential biomarkers. We detected 206 differential genes in the GSE36961 dataset and 2919 differential genes in the GSE180313 dataset. Subsequently, 6 and 17 modules were generated in the WGCNA. Among them, the green, grey, and turquoise modules showed the highest correlation with HCM, and we selected them for further analysis. We also performed clustering in the GSE181764 dataset. According to the results of the LASSO model, RTN4, IER3, and COL4A1 were finally identified as potential biomarkers in HCM. These three key genes showed differential expression in the HCM-normal group comparison in the validation datasets GSE68316 and GSE32453. RTN4 was underexpressed in HCM, while IER3 and COL4A1 were overexpressed.

RTN4 (reticulon 4) affects nuclear membrane expansion, formation of nuclear pore complexes, and proper localization of inner membrane proteins [12]. It regulates endoplasmic reticulum (ER) membrane morphogenesis by promoting the generation of tubular endoplasmic reticulum [13], inducing the formation and stability of ER tubules [14], and regulating lipid homeostasis [15]. In diseases related to the heart, RTN4 has been shown to be involved in the regulation of coronary heart disease [16] and is a key indicator of heart failure [17]. Its expression has been found to be upregulated in animal models of HCM [18]. RBM20 (RNA binding motif protein 20) acts as a splicing repressor, regulating the exons of RTN4 [19]. The expression of RBM20 mRNA in the left ventricle of HCM patients is significantly higher than that in normal controls, and the high expression of RBM20 mRNA is associated with a higher susceptibility to HCM. Its mechanism may involve the 12 rare deleterious variants present in RBM20, which may play a role in causing protein dysfunction and increasing the risk of sudden cardiac arrest (SCA) in HCM [20]. In HCM, the high expression of splicing repressor RBM20 inhibits the exons of RTN4, affecting the gene expression of RTN4 and reducing its expression. Therefore, the expression level of RTN4 can reflect the expression of RBM20 and has a predictive role in the occurrence and development of HCM.

IER3 (radiation-inducible immediate-early gene IEX-1) may play a role in the ERK signaling pathway by inhibiting the phosphatase PP2A-PPP2R5C to dephosphorylate ERK [21, 22]. It also mediates survival effects as a downstream effector of ERK [23, 24]. In experimental heart failure, the IER3 protein targets the promoter of anti-apoptotic genes and acts as an anti-apoptotic factor in myocardial cells. Proper activation of IER3 expression in the heart may be involved in cardiac remodeling processes, which play a key role in HCM. In a mouse model, biomechanical strain activated NF-kB in cultured cardiac myocytes, and iex-1 (IER3) served as a biomarker for NF-kB [25]. iex-1 is a mechanosensitive gene in myocardial cells and its product is involved in the hypertrophic response to mechanical strain. Overexpression of iex-1 can eliminate cardiomyocyte hypertrophy through mechanical strain, epinephrine, or endothelin-1, without affecting cell viability [26]. In HCM, due to pathological or physiological factors leading to cardiomyocyte hypertrophy and mechanical force changes, high expression of IER3 is needed to eliminate cardiomyocyte hypertrophy and participate in the regulation of myocardial cell mechanical force, restoring normal physiological morphology and function of cardiac muscle cells. Therefore, IER3 can serve as a biomarker for observing cardiomyocyte hypertrophy in HCM.

COL4A1 (collagen alpha-1(IV) chain) captures structures containing the C-terminal NC1 domain, inhibiting angiogenesis and tumor formation [27, 28]. The C-terminal half is found to have anti-angiogenic activity, specifically inhibiting endothelial cell proliferation, migration, and tube formation [29]. A study found that downregulation of COL4A1 in smooth muscle cells and endothelial cells increased apoptosis, impairing normal cardiac vascular function and increasing the risk of coronary heart disease [30]. Coronary artery injury leads to myocardial cell hypoxia [31], and hypoxia-inducible factor (HIF) 2*α* has a close relationship with the development of hypertrophic cardiomyopathy. Chronic activation of HIF-2*α* causes local inflammation in adipose tissue, increases the production of circulating proinflammatory and chemotactic factors, and then induces hypertrophic cardiomyopathy by activating the NF*κ*B and NFAT pathways [32]. In HCM, high expression of COL4A1 may be aimed at reducing apoptosis in cardiac endothelial and smooth muscle cells, improving the physiological state of cardiomyocyte hypoxia, reducing the stimulation of hypoxia-inducible factor on HCM, and serving as a potential biomarker for responding to the hypoxic condition of myocardial cells to observe the development of HCM.

By comparing with Maraini et al.'s study on inherited arrhythmias in pediatric populations, we found some commonalities and differences in the roles of RTN4, RBM20, and IER3 in heart diseases, in terms of genetic basis and pathogenic mechanisms [33]. For example, studies show that RTN4 plays a key role in regulating coronary heart disease, and Maraini et al.'s study found that RTN4 also plays an important role in inherited arrhythmias in pediatric populations. In addition, RBM20, as a splicing repressor, may have a similar mechanism in high expression in HCM and inherited arrhythmias in pediatric populations, warranting further exploration of its link between the two diseases. On the other hand, the critical role of IER3 in heart failure may intersect with the development of pediatric arrhythmias, suggesting that potential common signaling pathways or genetic variations exist. By comparing the consistencies and differences, we can more comprehensively understand the mechanisms of action of these genes in different heart diseases, providing deeper insight for further research and clinical practice.

## 5. Conclusion

Among the main pathogenic genes in HCM, MYH7 and MYBPC3 induce HCM mainly by influencing the gene structure domains encoding sarcomeres and sarcomere-associated proteins. Mutations in MYH7 have a significant impact on the globular head and hinge regions of myosin heavy chain proteins, leading to changes in the rod structure domain [34, 35]. MYBPC3 mutations are mainly nonsense and frameshift mutations, resulting in unstable or incorrectly folded proteins, which are subsequently degraded by the ubiquitin-proteasome system [36–38]. Through WGCNA and LASSO model, this study identified RTN4, COL4A1, and IER3 as potential biomarkers for responding to the occurrence and development of HCM, suggesting that the pathogenesis of HCM may be related to protein disruption, mechanical stress, and hypoxia. The identified biomarkers and mechanisms in this study may provide valuable information for further understanding the pathogenesis of HCM and for the improvement of HCM treatment, diagnosis, counseling, and prevention [39–43].

### 5.1. Limitations

Although valuable insights have been gained from this study, there are some limitations to consider. This study primarily focused on bioinformatics analysis of hypertrophic cardiomyopathy (HCM), emphasizing the genetic and molecular mechanisms of the disease. However, these findings are based on computational analysis and bioinformatics tools, requiring further experimental validation to confirm the identified genes and pathways in the pathogenesis of HCM. Additionally, the use of public datasets introduces potential biases that may affect the generalizability of the study results, and inherent limitations in data quality, completeness, and accuracy may affect the robustness of the conclusions drawn from the analysis. Furthermore, factors such as the sensitivity and specificity of algorithms and the quality of input data can affect the reliability of bioinformatic predictions. Additionally, the identification of RTN4, COL4A1, and IER3 as potential biomarkers associated with the development of HCM provides valuable insights into the molecular features of the disease. However, it must be recognized that the biomarkers and mechanisms elucidated in this study need to be validated in independent cohorts and functional experiments to determine their robustness and specificity. While the findings of this study provide a promising outlook for advancing the diagnosis, treatment, and prevention strategies for HCM, it is important to recognize the inherent limitations of bioinformatics analysis and the need for a multidisciplinary approach that integrates computational results with experimental validation to unravel the complexity of HCM pathophysiology.

## Data Availability

The materials and methods dataset provided by Hebl et al. for GSE36961, Li et al. for GSE89714, Ranjbarvaziri et al. for GSE180313, Wu et al. for GSE68316, and Gennebäck et al. for GSE32453 was obtained from the GEO database (https://www.ncbi.nlm.nih.gov/geo/).

## Supplementary Materials

Supplementary Materials Supplementary Table 1: baseline analysis of demographic information (age and gender) of RTN4, IER3, and COL4A1.
