# Supplementary material for: Exploring Hypertrophic Cardiomyopathy Biomarkers through Integrated Bioinformatics Analysis: Uncovering Novel Diagnostic Candidates
Source: Cardiol Res Pract. 2024 Jul 4;2024:4639334. doi: 10.1155/2024/4639334 (PMC11239233; doi:10.1155/2024/4639334)
Supplement: Supplementary Materials — Supplementary Table 1: baseline analysis of demographic information (age and gender) of RTN4, IER3, and COL4A1. [file 4639334.f1.pdf]

AGE

Baseline Table

group

Variable	Overall, N = 145[1]	age (yrs): 10, N = 2[1]	age (yrs): 11, N = 1[1]	age (yrs): 13, N = 1[1]	age (yrs): 14, N = 1[1]	age (yrs): 15, N = 4[1]	age (yrs): 16, N = 2[1]	age (yrs): 17, N = 2[1]	age (yrs): 19, N = 3[1]	age (yrs): 20, N = 1[1]	age (yrs): 21, N = 3[1]	age (yrs): 23, N = 4[1]	age (yrs): 25, N = 1[1]	age (yrs): 26, N = 2[1]	age (yrs): 27, N = 3[1]	age (yrs): 28, N = 1[1]
**IER3, Median (IQR)**	3.369 (3.284 – 3.431)	3.371 (3.336 – 3.406)	3.493 (3.493 – 3.493)	3.468 (3.468 – 3.468)	3.459 (3.459 – 3.459)	3.446 (3.436 – 3.477)	3.378 (3.368 – 3.388)	3.308 (3.208 – 3.409)	3.345 (3.296 – 3.420)	3.412 (3.412 – 3.412)	3.384 (3.325 – 3.414)	3.353 (3.284 – 3.419)	3.260 (3.260 – 3.260)	3.435 (3.400 – 3.469)	3.350 (3.305 – 3.430)	3.315 (3.315 – 3.315)
**COL4A1, Median (IQR)**	3.566 (3.522 – 3.617)	3.650 (3.608 – 3.691)	3.695 (3.695 – 3.695)	3.682 (3.682 – 3.682)	3.630 (3.630 – 3.630)	3.655 (3.634 – 3.667)	3.647 (3.612 – 3.681)	3.610 (3.589 – 3.630)	3.620 (3.560 – 3.647)	3.448 (3.448 – 3.448)	3.512 (3.503 – 3.528)	3.577 (3.553 – 3.604)	3.628 (3.628 – 3.628)	3.601 (3.598 – 3.604)	3.542 (3.522 – 3.635)	3.585 (3.585 – 3.585)
**RTN4,**	3.362	3.375	3.257	3.419	3.397	3.393	3.327	3.389	3.337	3.385	3.281	3.380	3.399	3.373	3.343	3.404

Variable	age (yrs): 30, N = 3[1]	age (yrs): 31, N = 2[1]	age (yrs): 32, N = 2[1]	age (yrs): 33, N = 1[1]	age (yrs): 35, N = 2[1]	age (yrs): 36, N = 1[1]	age (yrs): 37, N = 4[1]	age (yrs): 38, N = 2[1]	age (yrs): 39, N = 1[1]	age (yrs): 4, N = 1[1]	age (yrs): 40, N = 1[1]	age (yrs): 41, N = 2[1]	age (yrs): 42, N = 1[1]	age (yrs): 43, N = 4[1]	age (yrs): 44, N = 3[1]	age (yrs): 45, N = 5[1]	age (yrs): 46, N = 2[1]	age (yrs): 47, N = 3[1]
**IER3, Median (IQR)**	3.430 (3.400 – 3.472)	3.272 (3.233 – 3.312)	3.425 (3.422 – 3.429)	3.367 (3.367 – 3.367)	3.499 (3.439 – 3.560)	3.219 (3.219 – 3.219)	3.290 (3.235 – 3.362)	3.369 (3.366 – 3.372)	3.155 (3.155 – 3.155)	3.124 (3.124 – 3.124)	3.139 (3.139 – 3.139)	3.176 (3.158 – 3.195)	3.409 (3.409 – 3.409)	3.404 (3.339 – 3.452)	3.401 (3.294 – 3.424)	3.327 (3.326 – 3.443)	3.341 (3.308 – 3.374)	3.418 (3.315 – 3.432)
**COL4A1, Median (IQR)**	3.572 (3.541 – 3.642)	3.557 (3.546 – 3.568)	3.593 (3.588 – 3.598)	3.680 (3.680 – 3.680)	3.603 (3.599 – 3.607)	3.550 (3.550 – 3.550)	3.614 (3.591 – 3.632)	3.587 (3.584 – 3.589)	3.622 (3.622 – 3.622)	3.555 (3.555 – 3.555)	3.516 (3.516 – 3.516)	3.499 (3.473 – 3.526)	3.517 (3.517 – 3.517)	3.579 (3.552 – 3.599)	3.580 (3.574 – 3.593)	3.657 (3.565 – 3.674)	3.627 (3.621 – 3.633)	3.692 (3.630 – 3.716)
**RTN4,**	3.325	3.408	3.388	3.321	3.420	3.296	3.390	3.410	3.344	3.398	3.239	3.323	3.255	3.320	3.380	3.332	3.351	3.422

Variable	age (yrs): 48, N = 5[1]	age (yrs): 49, N = 1[1]	age (yrs): 50, N = 2[1]	age (yrs): 51, N = 4[1]	age (yrs): 52, N = 5[1]	age (yrs): 53, N = 4[1]	age (yrs): 54, N = 4[1]	age (yrs): 55, N = 2[1]	age (yrs): 56, N = 4[1]	age (yrs): 57, N = 1[1]	age (yrs): 58, N = 2[1]	age (yrs): 59, N = 6[1]	age (yrs): 60, N = 2[1]	age (yrs): 61, N = 1[1]	age (yrs): 62, N = 1[1]	age (yrs): 63, N = 1[1]	age (yrs): 64, N = 1[1]
**IER3, Median (IQR)**	3.292 (3.110 – 3.455)	3.117 (3.117 – 3.117)	3.305 (3.283 – 3.327)	3.306 (3.194 – 3.362)	3.425 (3.213 – 3.499)	3.229 (3.188 – 3.316)	3.322 (3.226 – 3.379)	3.267 (3.201 – 3.332)	3.312 (3.308 – 3.322)	3.425 (3.425 – 3.425)	3.459 (3.444 – 3.474)	3.421 (3.395 – 3.479)	3.364 (3.349 – 3.379)	3.114 (3.114 – 3.114)	3.371 (3.371 – 3.371)	3.292 (3.292 – 3.292)	3.529 (3.529 – 3.529)
**COL4A1, Median (IQR)**	3.562 (3.522 – 3.564)	3.660 (3.660 – 3.660)	3.590 (3.559 – 3.620)	3.496 (3.475 – 3.533)	3.534 (3.494 – 3.558)	3.548 (3.510 – 3.586)	3.514 (3.500 – 3.528)	3.586 (3.582 – 3.590)	3.494 (3.481 – 3.517)	3.475 (3.475 – 3.475)	3.606 (3.596 – 3.615)	3.556 (3.554 – 3.602)	3.514 (3.503 – 3.524)	3.610 (3.610 – 3.610)	3.554 (3.554 – 3.554)	3.505 (3.505 – 3.505)	3.555 (3.555 – 3.555)
**RTN4,**	3.379	3.500	3.384	3.310	3.285	3.355	3.363	3.436	3.379	3.262	3.277	3.211	3.358	3.440	3.322	3.412	3.302

Variable	age (yrs): 65, N = 3[1]	age (yrs): 66, N = 1[1]	age (yrs): 67, N = 6[1]	age (yrs): 69, N = 2[1]	age (yrs): 7, N = 1[1]	age (yrs): 70, N = 2[1]	age (yrs): 71, N = 3[1]	age (yrs): 73, N = 2[1]	age (yrs): 75, N = 1[1]	age (yrs): 76, N = 1[1]	age (yrs): 77, N = 1[1]	age (yrs): 78, N = 1[1]	age (yrs): 9, N = 1[1]	tissue: cardiac, N = 3[1]	p-value[2]
**IER3, Median (IQR)**	3.322 (3.285 – 3.331)	3.371 (3.371 – 3.371)	3.401 (3.369 – 3.480)	3.354 (3.332 – 3.376)	3.141 (3.141 – 3.141)	3.375 (3.365 – 3.384)	3.412 (3.394 – 3.413)	3.434 (3.399 – 3.470)	3.448 (3.448 – 3.448)	3.316 (3.316 – 3.316)	3.429 (3.429 – 3.429)	3.318 (3.318 – 3.318)	3.360 (3.360 – 3.360)	3.284 (3.185 – 3.322)	0.427
**COL4A1, Median (IQR)**	3.491 (3.460 – 3.536)	3.665 (3.665 – 3.665)	3.546 (3.538 – 3.562)	3.585 (3.573 – 3.596)	3.591 (3.591 – 3.591)	3.571 (3.538 – 3.605)	3.514 (3.499 – 3.533)	3.549 (3.536 – 3.561)	3.515 (3.515 – 3.515)	3.425 (3.425 – 3.425)	3.561 (3.561 – 3.561)	3.532 (3.532 – 3.532)	3.629 (3.629 – 3.629)	3.676 (3.579 – 3.703)	0.054
**RTN4,**	3.251	3.283	3.365	3.372	3.244	3.411	3.361	3.400	3.337	3.377	3.317	3.373	3.408	3.422	0.368

[1] Median (IQR) or Frequency (%)

[2] Kruskal-Wallis rank sum test

GENDER

Baseline Table

group

Variable	Overall, N = 145[1]	Sex: female, N = 72[1]	Sex: male, N = 73[1]	p-value[2]
**IER3, Median (IQR)**	3.369 (3.284 – 3.431)	3.369 (3.280 – 3.420)	3.369 (3.287 – 3.444)	0.79
**COL4A1, Median (IQR)**	3.566 (3.522 – 3.617)	3.568 (3.521 – 3.616)	3.565 (3.524 – 3.617)	0.719
**RTN4, Median (IQR)**	3.362 (3.307 – 3.404)	3.372 (3.318 – 3.410)	3.357 (3.281 – 3.399)	0.149

[1] Median (IQR) or Frequency (%)

[2] Wilcoxon rank sum test
